# Exploring Zinc-Doped Manganese Hexacyanoferrate as Cathode for Aqueous Zinc-Ion Batteries

**DOI:** 10.3390/nano14131092

**Published:** 2024-06-25

**Authors:** Julen Beitia, Isabel Ahedo, Juan Ignacio Paredes, Eider Goikolea, Idoia Ruiz de Larramendi

**Affiliations:** 1 Departamento de Química Orgánica e Inorgánica, Universidad del País Vasco (UPV/EHU), Barrio Sarriena s/n, 48940 Leioa, Spain; julen.beitia@ehu.eus (J.B.); isabel.ahedo@ehu.eus (I.A.); eider.goikolea@ehu.eus (E.G.); 2 Instituto de Ciencia y Tecnología del Carbono, INCAR-CSIC, C/Francisco Pintado Fe 26, 33011 Oviedo, Spain; paredes@incar.csic.es

**Keywords:** zinc, Prussian Blue analogue, cathode, aqueous battery

## Abstract

Aqueous zinc-ion batteries (AZiBs) have emerged as a promising alternative to lithium-ion batteries as energy storage systems from renewable sources. Manganese hexacyanoferrate (MnHCF) is a Prussian Blue analogue that exhibits the ability to insert divalent ions such as Zn^2+^. However, in an aqueous environment, MnHCF presents weak structural stability and suffers from manganese dissolution. In this work, zinc doping is explored as a strategy to provide the structure with higher stability. Thus, through a simple and easy-to-implement approach, it has been possible to improve the stability and capacity retention of the cathode, although at the expense of reducing the specific capacity of the system. By correctly balancing the amount of zinc introduced into the MnHCF it is possible to reach a compromise in which the loss of capacity is not critical, while better cycling stability is obtained.

## 1. Introduction

Nowadays, one of the most critical challenges in our society is to enhance the use of natural and renewable energy sources that are almost inexhaustible and do not generate any environmental degradation. The most important renewable energies are solar, wind, marine, and geothermal. As an advantage, these alternative energy forms allow society to obtain substantial amounts of energy without polluting the environment with greenhouse gas emissions among others. However, these sources depend on geography, climate, and time of day, so it is important to be able to store energy surpluses in an efficient way that allows their use later when the production goes down [1,2,3]. To overcome the problem related to their intermittent nature, research efforts should also focus on the development of new energy storage systems that help managing the demand/supply dynamics. 

Among the various energy storage systems, batteries are particularly important. These systems can store energy in the form of a chemical reaction and later convert it back into electric current. Lithium ion batteries (LIBs) were successfully marketed by SONY in 1991, and ever since then, this battery technology has stood out for its remarkable gravimetric and volumetric energy densities and excellent cycle life compared to other rechargeable batteries. However, this technology has a number of significant drawbacks such as the limited access to lithium reserves, the high cost of the components, and safety concerns related to the flammable nature of organic electrolytes [4]. Moreover, the massive and rapid growth of the electric vehicle market is incentivising the development of alternative technologies to LIBs, not only to meet the needs of the portable energy storage market but also those of stationary applications.

In recent years research has focused on aqueous systems as an alternative to lithium-based systems due to the need for environmentally friendly, safe, and cost-effective devices [5,6]. Therefore, battery systems operating in an aqueous electrolyte are preferable to those working in organic ones. Aqueous electrolytes exhibit interesting advantages such as high ionic conductivity, in addition to being easier to assemble since the need of a controlled atmosphere is eliminated. Among the different aqueous systems, aqueous zinc-ion batteries (AZiBs) stand out for their unique properties linked to the high abundance of metallic zinc, eco-friendliness, intrinsic safety, and cost-effectiveness, making them a promising choice for large-scale renewable energy storage applications [7,8].

AZiBs use metallic zinc in the form of a foil or as powder forming composite electrode, which will oxidize throughout the discharge releasing Zn^2+^ ions. These ions will pass through the near-neutral (or mildly acidic) aqueous electrolyte reaching the cathode where the Zn^2+^ ions will be reversibly inserted into the active material, thanks to the reduction of some electroactive elements that make it up. Throughout the charge, the reverse process occurs, with zinc being electrodeposited on the surface of the anode. During cycling, some issues can occur which are due to inhomogeneous deposition, which can form metallic Zn dendrites and short-circuit the system by the perforation of the separator or the release of hydrogen in the anode (Hydrogen Evolution Reaction, HER) which is formed in the potentials where Zn is electrodeposited. It should be noted that the formation of this gaseous hydrogen is one of the most important issues in the development of AZiBs and needs to be studied in more detail. Recent studies have shown that the control of gas release is possible through control of the pH of the electrolyte or using additives in solution [9,10].

Despite certain issues that still need to be solved, the commercialization of these Zn-ion-based batteries becomes feasible as a suitable, cheap, and environmentally friendly system having high capacity and stability in the long term [11,12]. The selection of a cathode with a high working voltage and a large specific capacity is a critical parameter to increase the gravimetric energy density of AZiBs and to be competitive with the LIBs [13]. In fact, the design and development of cathode materials with high storage capacity, high discharge potential, and a robust crystalline structure with easy insertion and removal pathways has been a great challenge in the development of high-performance AZiBs. Among the most studied materials, oxides such as MnO_2_ or V_2_O_5_ and Prussian Blue analogues (PBAs) are found. Concerning the oxides, MnO_2_ presents a good cycle life, but the low ionic and electronic conductivities limit its use, and in the case of vanadium-based oxides, promising capacities and good cycle life are reached but their low operating potential leads to low energy density of the final system [14,15,16]. The mixed-valence hexacyanoferrate family (Prussian Blue and its analogues) is another group of materials that is of special interest as cathodes in AZiBs. Their open structure, combined with the wide variety of metals that can be part of the structure, allows the electrochemical properties of the material to be tuned [17,18]. These phases have high working potentials, although their specific capacity is limited and they present poor cycling stability [13]. 

**Figure 1:**
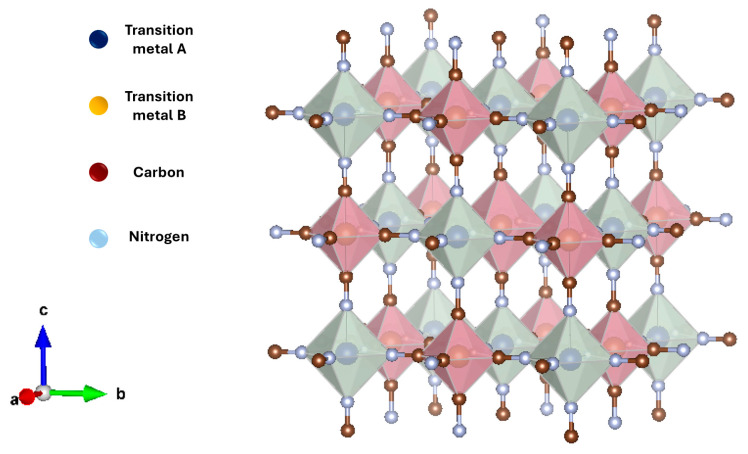
Structure model of a Prussian Blue analogue (PBA).

PBAs have attracted attention in the last few years in the field of energy storage due to their easy and inexpensive synthesis procedure by coprecipitation, high specific capacity for the reversible insertion of metallic ions, their high safety and nontoxicity, and the electrochemical properties that can be tuned through the variation of the material composition [19,20]. These materials came from the ancient discovered blue pigment Prussian Blue (PB; Fe_4_[Fe(CN)_6_]_3_) which is one of the oldest synthetic coordination polymers. Over the last century, PB has been modified by doping the iron sites with other transition metals such as Mn, Ni, Co, or Zn among others, to obtain analogues (PBAs) and meet different application and research requirements [19,21]. The general chemical formula of PBAs is A_a_TM_A_[TM_B_(CN)_6_]_n_ · xH_2_O where A is the alkali metal ion that can be sodium (Na^+^) or potassium ions (K^+^), while TM_A_ and TM_B_ are the transition metal ions which replace iron in PB. In the structure of PBAs (see Figure 1), TM_A_ is coordinated to the nitrogen atom of the cyanide ion and TM_B_ is surrounded by the carbon atom of the cyanide ion. This coordination of the cyanide group makes PBAs compounds exhibit a 3D open structure with many interstitial sites where zinc ions can be reversely inserted in the battery cycling process. 

Among the wide variety of possible PBAs, one of the most studied families is the manganese-based hexacyanoferrates (MnHCF) since it benefits from the existence of two redox couples (Fe^3+^/Fe^2+^ and Mn^3+^/Mn^2+^) delivering large specific capacities [17,18,22]. The charge storage mechanism through Zn^2+^ insertion/extraction in AZiBs using MnHCF as cathodes follows the reaction (1):

x Zn^2+^ + MnHCF + 2x e^−^ ⇌ Zn_x_MnHCF (1)

During the discharge, the Zn^2+^ ions produced in the oxidation of the zinc anode are inserted into the MnHCF structure through the reduction of the two active redox couples. During charging, the reverse process occurs, returning the Zn^2+^ ions extracted from the cathode to the anode where they will be reduced to metallic zinc. The amount of zinc that can be reversibly inserted depends on many factors such as water content or particle size, among others. The main problem that MnHCF faces is related to its structural instability in aqueous medium. Although in the first cycles, MnHCF is capable of providing specific capacities as high as 140 mAh g^−1^ (at current densities of 100 mA g^−1^) [23], the material suffers from structural instability upon cycling, forming new zinc-rich phases and, ultimately, dissolving the cathode material [24]. To avoid these disadvantages, different approaches have been investigated such as the preparation of MnHCFs anchoring MnO_2_ [25], the design of hybrid systems such as MnHCF/polymer [23,26], the nanostructuring of the MnHCF through the use of surfactants during the synthesis [27], or adjusting the electrolyte formulation [28,29]. In this work, a different strategy is explored that is based on doping the MnHCF phase with zinc in the manganese position. In this way, the aim is to provide higher structural stability to the material that allows its implementation in AZiBs. This approach allows the preparation of materials through a simple and environmentally friendly synthetic route. 

In this work, electrochemical behaviour in Zn chemistry of K(Mn_1−x_Zn_x_)[Fe(CN)_6_] (x = 0, 0.25, 0.5, 0.75, and 1) PBAs is studied. This new family of zinc-doped manganese-based PBAs was obtained by coprecipitation through controlling the dropping speed and the reactants’ concentration. The structure and morphology of the obtained materials were studied in order to examine the effect of zinc doping. Also, one of the most important goals of this work is to be able to optimize the performance of each of the systems that are used, and testing and finding the most suitable material to achieve greater electrochemical performance. The new family of zinc-doped manganese-based PBAs will be obtained by the coprecipitation method through controlling the dropping speed and the reactants’ concentration. The structure and morphology of the obtained materials will be studied in order to examine the effect of zinc doping. Finally, the electrochemical performance of the materials will be examined delving into the role of zinc on the behavior of MnHCF and its structural stability.

## 2. Materials and Methods

### 2.1. Synthesis of the PBA Compounds 

The synthesis of K(Mn_1−x_Zn_x_)[Fe(CN)_6_] (x = 0, 0.25, 0.5, 0.75, and 1), or Mn_1−x_Zn_x_HCF, compounds was carried out by coprecipitation method. For the synthesis, stoichiometric quantities of the reagents were mixed as detailed in Table 1. In this regard, ZnSO_4_ (Sigma-Aldrich, St. Louis, MO, USA) and Mn(NO_3_)_2_ (Sigma-Aldrich, St. Louis, MO, USA) were separately dissolved in distilled water (100 mL) at room temperature. On the other hand, another solution was simultaneously prepared dissolving K_3_Fe(CN)_6_ (0.002 mol, Sigma-Aldrich, St. Louis, MO, USA) in distilled water (100 mL) at room temperature too. Once all the reagents were completely dissolved, PBAs were synthesized by mixing dropwise the solutions (Figure S1), which gave a white-coloured solid in suspension in a solution with a pH of 5–6. 

### 2.2. Physico-Chemical Characterization of the PBAs

The identification and the structural characterization of the compounds were carried out by X-ray diffraction (XRD, Panalytical X’Pert PRO) between 5 and 70° (2θ) using Cu radiation (Cu-K_αa_ λ = 0.15418 nm). Also, attenuated total reflection infrared spectroscopy (FTIR) was performed using Shimadzu FTIR-8400S (Kyoto, Japan) equipment to analyse directly the pre-cycling materials without any pre-treatment. The morphology of the initial samples was studied by scanning electron microscopy (SEM, Hitachi S-4800, Tokyo, Japan).

### 2.3. Preparation of Electrodes

The positive electrode composition was 70 wt.% PBA, 20 wt.% conductive Ketjen black carbon, and 10 wt.% polytetrafluoroethylene; PTFE, from a 60 wt.% dispersion in H_2_O (Sigma-Aldrich, St. Louis, MO, USA). Negative electrode composition was 99.9 wt.% trace zinc (thickness 0.25 mm, Sigma-Aldrich, St. Louis, MO, USA). This mixture was drenched in ethanol and blended until the plasticity was good enough to obtain a thin black film (150 µm) on carbon paper (H23C6; Quintech Brennstoffzellen Technolgie, Göppingen, Germany). This thin film was left to dry in a vacuum oven at 80 °C for 24 h, and after drying the laminates were cut into 11 mm diameter circular electrodes and finally, weighed and labelled.

### 2.4. Electrochemical Characterization

The different samples were electrochemically tested using a multichannel potentiostat/galvanostat VMP3 (BioLogic, Seyssinet-Pariset, France) performing cyclic voltammetry measurements at scan rates of 0.1, 0.2, 0.3, 0.5, 0.8, and 1 mV s^−1^. Galvanostatic charge/discharge measurements using different current densities of 0.1, 0.2, 0.3, 0.5, 1, and 2 A g^−1^ were performed between 0.005–2.0 V vs. Zn^2+^/Zn. 

In the case of the cyclic voltammetry measurements, the assembly of the electrochemical cells was carried out in three-electrode Swagelok^®^ cell systems (Swagelok^®^, Solon, OH, USA) using stainless steel current collectors, Mn_1−x_Zn_x_HCF-based electrodes as working electrodes, metallic zinc disc as the counter, a Ag/AgCl in saturated KCl as the reference electrode, and a porous glass fibre (Whatman GF/A, Maidstone, UK) membrane as the separator with a 3 M zinc trifluoromethanesulfonate (Zn(OTf)_2_) (Sigma-Aldrich, St. Louis, MO, USA) in distilled water as the electrolyte.

For galvanostatic measurements, Mn_1−x_Zn_x_HCF compounds were tested in two-electrode Swagelok^®^ cell systems (Swagelok^®^, Solon, OH, USA) using stainless steel current collectors, a metallic zinc disc as both the counter and the reference electrode and a porous glass fibre (Whatman GF/A, Maidstone, UK) membrane as the separator. In all cases, 3 M zinc trifluoromethanesulfonate (Zn(OTf)_2_) (Sigma-Aldrich, St. Louis, MO, USA) dissolved in distilled water was used as the electrolyte.

## 3. Results and Discussion

**Figure 2:**
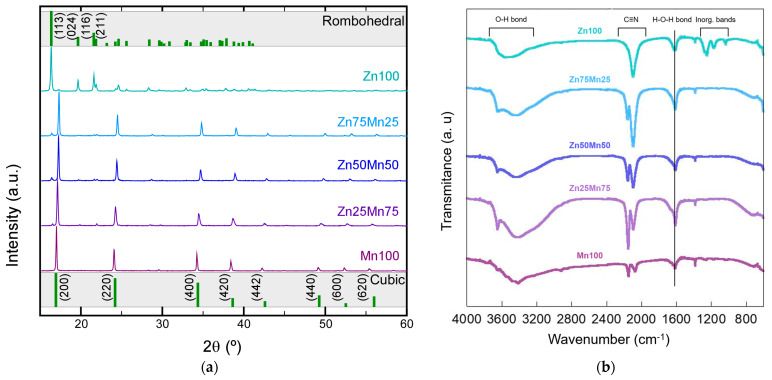
(**a**) XRD patterns and (**b**) infrared spectra of Mn_1−x_Zn_x_HCF (x = 0, 0.25, 0.5, 0.75, and 1) samples.

A new family of PBAs with different Zn and Mn contents, Mn_1−x_Zn_x_HCF (x = 0, 0.25, 0.5, 0.75, and 1), has been synthesised and characterised. PBAs can crystallise in different crystal structures depending on factors such as the synthesis process, the type of transition metal, or the degree of zinc insertion. Among the most common structures in the literature cubic, orthorhombic, rhombohedral, and monoclinic are found [30,31,32]. Figure 2a shows the diffractograms obtained for the synthesised samples together with the theoretical positions of the main diffraction peaks for the cubic (space group: *Fm*-3*m*) and rhombohedral (space group: *R*-3*c*) structures. Using the coprecipitation synthesis route, in the case of the manganese sample (Mn100), the diffraction peaks are well-defined and sharp and the diffraction pattern matches with the cubic structure, in good agreement with the structure reported by other authors in the literature [33,34,35,36,37]. The diffraction pattern of the Zn100 sample fits a rhombohedral/hexagonal structure (*R*-3*c*), as has been described in the bibliography [35,38,39], and no impurities are observable. The samples doped with Zn in the Mn site present a diffraction pattern corresponding to a cubic structure, although as the Zn content increases, the presence of a small peak around 16.2° (2θ), accompanied by other weak signals at higher angles can be detected. This new peak appears at an angle very close to the 100% intensity peak of the rhombohedral phase corresponding to the Zn100 sample. The presence of this peak can be explained based on the presence of a small concentration of a secondary phase with a rhombohedral structure together with a preferential phase with a cubic structure. 

In this way, the effect of zinc is evident, distorting the cubic structure that these types of compounds usually exhibit, although zinc-based PBAs with cubic structure have also been reported [40]. The type of structure exhibited by these types of compounds is highly dependent on the synthesis conditions [40,41]. A slight change in the preparation temperature or the pH of the medium can easily cause a change in the structure. In order to avoid this type of effect, all the samples in this work have been synthesised under the same conditions, trying to avoid producing structural or morphological changes beyond those imposed by the change in phase composition.

Going on with the structural study, the analysis and interpretation of the FTIR spectra presented in Figure 2b have been carried out [42,43,44]. The recorded spectra show one major signal in the 3700–3000 cm^−1^ range related to the H_2_O bands (H-O tensile vibration peaks), typical of these compounds [34]. This indicates the presence of water molecules, surely on the surface and even in the open sites of the structure of the compounds. In the central part of the infrared spectrum, between 2300 and 1200 cm^−1^, four signals can be distinguished. The peaks around 2100 cm^−1^ correspond to the cyanide group (C≡N) and the peak around 1600 cm^−1^ is the H-O-H bond bending vibrations of water molecules [43]. Finally, in the region between 1400 and 1000 cm^−1^, the bands related to inorganic compounds are observed, and in some cases, in the zone between 1000 and 500 cm^−1^, the bands corresponding to functional groups bond to the metal can be detected. In this case, two signals that correspond to the bonding of the metal with the cyanide group are observed. From the analysis of the spectra, two important features are highlighted. On the one hand, the existence of signals clearly assignable to water molecules makes clear the presence of water in the compounds. On the other hand, the change in the intensity ratio of the bands attributed to the stretching vibration peak of the bridging cyano group (–CN), suggests a change in the coordination environment of this group depending on the Zn/Mn ratio. In the Zn100 sample, a single peak can be seen that, as the amount of Mn increases, loses intensity, and a new peak appears at slightly higher wavenumber values (blue shift). The positions of the bands corresponding to the cyano group assigned to the Fe^III^—CN—M^II^ and Fe^II^—CN—M^II^ chains are around 2160 and 2080 cm^−1^, respectively, for the samples that have manganese in their composition. In the case of the Zn100 phase, a single band relative to the Fe^II^-C≡N-M^III^ bonds is observed, located at 2100 cm^−1^ [45,46]. The shift appreciated in the bands is due to the change in the electronic state of the cations linked to the N, which undergo a transition from high-spin (MII) to low-spin (MIII) [47].

**Figure 3:**
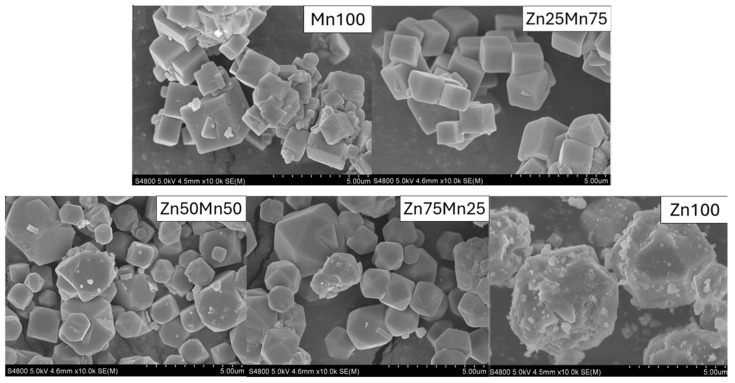
SEM images of Mn_1−x_Zn_x_HCF (x = 0, 0.25, 0.5, 0.75 and 1) samples.

The effect of Zn doping at the Mn site on the morphology of the materials was analyzed by SEM and the recorded images are shown in Figure 3. The Mn100 sample exhibits a cubic shape with particles of different sizes, between 50 nm and 3 μm, as has been widely reported in the literature [26,33,48,49]. The solubility constant of the K_2_Mn[Fe(CN)_6_] phase (K_sp_ = 10^−12.1^) is very low [50], which causes this compound to precipitate very easily and quickly in solution, giving rise to particles without a defined shape. When the precipitation process is controlled by adding, for example, chelating agents or slowing down the formation of the product, it is possible to obtain cubic-shaped particles with few defects [51]. In the synthesis process of the PBAs presented in this work, special care has been taken to control the reaction time, adding the reagents simultaneously and slowly, drop by drop, in order to control the nucleation and growth of the particles in an efficient and simple way. In this case, it is critical to control the synthesis conditions since they have a great impact both on the structure of the compound and on the size and morphology of the particles. Lee and Huh already demonstrated that by increasing the concentration of HNO_3_ in the synthesis of KFe^III^[Fe^II^(CN)_6_, the particle morphology evolved from cubes to star-like hexapods [41]. This morphological change allowed them to determine that the oxidation processes began in the corners of the cubes, which act as active sites. Zhang et al. carried out a similar study on zinc hexacyanoferrate, and by adjusting the concentration of reagents and controlling the dropping speed, particles with different polyhedral shapes were prepared: cuboctahedrons, truncated octahedron, and octahedron zinc hexacyanoferrates [40]. In fact, it has been reported that in the case of certain zinc-based PBAs, the cubic structure is not stable, since water molecules can leave the structure relatively easily, giving rise to a transformation towards a rhombohedral phase [52,53]. In our case, Zn100 particles show a cuboctahedral shape, very different from that of Mn100 particles. Furthermore, the particles appear to be covered with small fragments, presenting rough surfaces, unlike the Mn100 sample in which the surfaces of the sides of the cubes are quite smooth. As the zinc ratio of the samples increases, the shape of the particles evolves from cubes to cuboctahedrons. As determined in the structural study, the samples tend to maintain the structure and shape imposed by manganese in the PBAs, but at high Zn contents, the compound is forced to readapt, taking on more importance the characteristics of the Zn100 compound. Thus, while the Zn25Mn75 sample is made up of cubic particles with an edge of around 100 nm, the particles of the Zn75Mn25 sample show a clearly cuboctahedral shape, with smooth and clean surfaces.

Indeed, the composition of the phases is vital to determine the electrochemical properties of the material, but it has been shown that the morphology of the particles also has an important impact on the performance of the cathode. Specifically, the cuboctahedral shape of the Zn-based PBAs provides larger discharge capacities at high rates, in addition to exhibiting greater stability in cycling [40]. This fact has been related to the high surface area that this shape of the particles presents. In the case of the compounds in this study, a change in the morphology of the particles is observed, although there does not seem to be an effect on the particle size with the zinc content.

**Figure 4:**
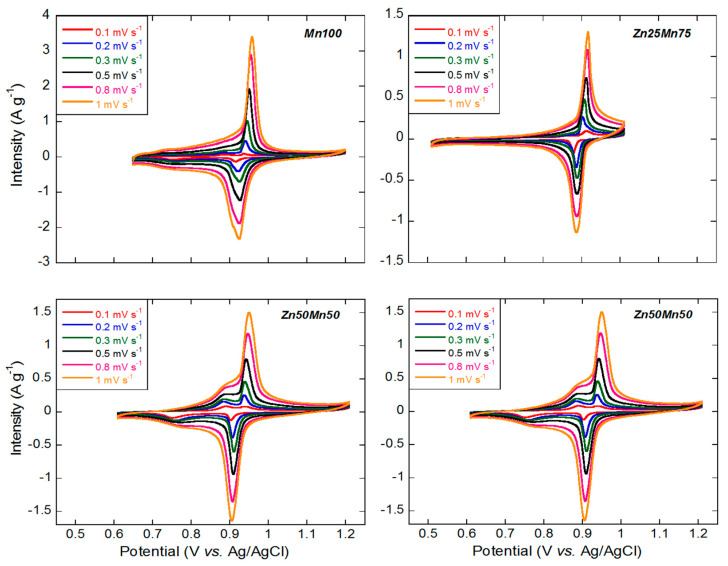
CV curves of the Mn_1−x_Zn_x_HCF (x = 0, 0.25, 0.5 and 0.75) samples at different scan rates: 0.1, 0.2, 0.3, 0.5, 0.8 and 1 mV s^−1^.

Before integrating the prepared materials into AZiBs, the reaction kinetics of each compound were studied by means of cyclic voltammetry (CV) measurements at different scan rates from 0.1 to 1.0 mV s^−1^. It should be noted that in the case of Zn100 sample, the CV obtained does not show any peak (Figure S2) and therefore its electrochemical activity is practically zero, in good agreement with results reported by other authors [54,55]. Also, as can be seen in Figure 4, in the case samples with high amounts of Mn (Mn100 and Zn25Mn75), CV curves show two major peaks, which maintain the shape during the experiment at different rates. The peaks that appear at 0.95/0.98 V vs. Ag/AgCl during oxidation and at ca. 0.90 V during reduction correspond to the Mn^3+^/Mn^2+^ redox pair and, as expected, as the scan rate increases the intensity of the peak increases, perceiving a slight shift towards higher potentials in the oxidation branch and towards lower values in the reduction branch. By increasing the zinc content (and therefore decreasing the amount of Mn in the sample), the appearance of another signal at 0.9/0.87 V vs. Ag/AgCl (oxidation) and ca. 0.75 V (reduction) is distinguished for the Zn50Mn50 phase that is assigned to the Fe^3+^/Fe^2+^ redox pair [39,56]. The intensity ratio of the redox pairs corresponding to Mn and Fe is further balanced when examining the sample Zn75Mn25. Taking into account the maximum intensities reached by the different samples, the superiority in terms of electrochemical activity of the Mn100 sample is clear.

In Figure S3 the relationship between the peak current (I_p_; mA g^−1^) and the square root of the used scan rates ($\sqrt{V}$; $\sqrt{mVs^{-1}}$) is represented [57]. This relationship is almost linear in all the samples, which indicates that all systems are compatible with and ideal diffusion-controlled faradaic process. Furthermore, the anodic and cathodic lines present great symmetry, which is indicative of the high reversibility of the redox process. Moreover, the peak separation at 0.1 mV s^−1^ is about 20 mV and increases just up to 35 mV at 1 mV s^−1^, which is also indicative of the reversibility of the reaction. 

Galvanostatic charge/discharge measurements were performed between 0.005–2.0 V vs. Zn^2+^/Zn using a current density of 0.1 A g^−1^. Figure S4 shows the evolution of the charge/discharge curves over 10 cycles for each sample. Again, it is possible to distinguish two different behaviours depending on the Zn content in the sample. On the one hand, the samples rich in manganese, Mn100 and Zn25Mn75, show 2–3 plateaus, being more defined in the case of the Mn100 sample. These small plateaus have been also reported in previous works using PBAs, specifically in works in which manganese has been used in this type of structure [58,59,60]. In the first discharge curves of the two samples with the highest Mn content, a small plateau is distinguished around 1.8 V vs. Zn^2+^/Zn that progressively disappears, being imperceptible from the seventh cycle onwards. Likewise, two other plateaus are distinguished between 0.8 and 1.5 V, the one with the highest potential being assigned to the Mn^3+/2+^ redox couple, while the one at lower potentials corresponds to the Fe^3+/2+^ redox couple, as was established in the analysis of CVs. The contribution to the capacity of both processes decreases upon cycling, with the plateau due to Fe^3+/2+^ disappearing in the last cycles. This change in the profiles, which ultimately become sloppy, may be related to structural changes that are usually related to the dissolution of Mn, leading to the formation of low crystalline MnO_2_ [61], and changes in the coordination environment of the transition metals in the structure [60]. When a higher amount of Zn is introduced into the structure (Zn50Mn50 and Zn75Mn25 samples), it is barely possible to distinguish the presence of well-defined plateaus; instead, a progressive drop is perceived. This type of profile differs from those reported by other authors analyzing zinc hexacyanoferrates in which only iron acts as an electrochemically active transition metal, distinguishing a plateau corresponding to the transference of one electron in sodium-based batteries [35]. Here, the presence of iron accompanied by manganese, which also presents electrochemical activity, produces a clear change in the profile. In any case, similar discharge profiles have been reported for zinc-doped hexacyanoferrates [26]. The profiles in-the-form slope in AZiBs have been justified based on the (in)stability of the PBAs, especially in the first cycles in which a structural readjustment occurs due to the deintercalation of the K^+^ cations [56]. Kim et al. analyzed the effect of the introduction of Zn in cobalt hexacyanoferrates, observing a similar transition from a cubic structure of the Co phase to a rhombohedral one as the Zn content increased [62]. The coexistence of both phases with different structures in the samples doped with Zn (mixture of cubic and rhombohedral phases observed in the study using XRD), together with the decrease in the content of electrochemically active metals causes a decrease in capacity, leading to a loss of activity observed in the case of the Zn100 phase. The presence of the rhombohedral phase in the samples with Zn facilitates higher stability in these compounds since the structural changes due to the (de)intercalation of Zn^2+^ ions in the cyclability are less severe [62]. This way, the stability of the material is higher, allowing for superior capacity retention. 

**Figure 5:**
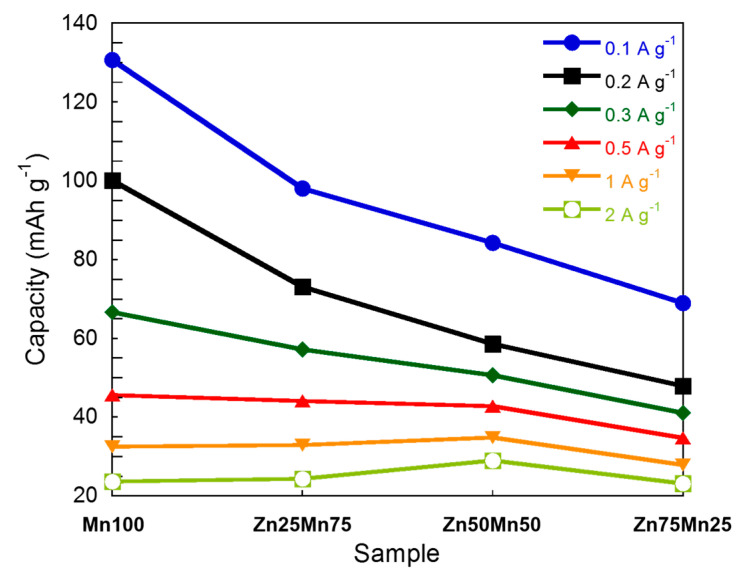
Capacity values at different currents for Mn_1−x_Zn_x_HCF (x = 0, 0.25, 0.5, and 0.75) samples.

In terms of electrochemical properties, the higher the Mn content is, the higher the capacity values are, as shown in Figure 5. In contrast, at high Mn contents, capacity values decrease significantly by the tenth cycle. For instance, for Mn100 sample capacity retention is 39% (130 mA h g^−1^ vs. 51 mA h g^−1^) while for Zn25Mn75 sample this retention is 53% (98 mA h g^−1^ vs. 52 mA h g^−1^). For Zn-enriched samples, instead, the capacity retention is much higher: 61% for Zn50Mn50 (64 mA h g^−1^ vs. 39 mA h g^−1^) and 94.5% for Zn75Mn25 (73 mA h g^−1^ vs. 69 mA h g^−1^). At low current intensities, the active material has enough time to fully charge and discharge, i.e., Zn^2+^ ions can intercalate and deintercalate into the PBA structure. As the current increases, diffusion processes and even charge transfer processes are hindered and the reaction yield is much lower. On the other hand, the decrease in capacity with increasing zinc content is related to the lack of electroactivity of Zn. The higher the concentration of Zn in the PBA structure, the lower the amount of Mn^3+/2+^ redox couple, which has a direct impact on the capacity of the compound.

**Figure 6:**
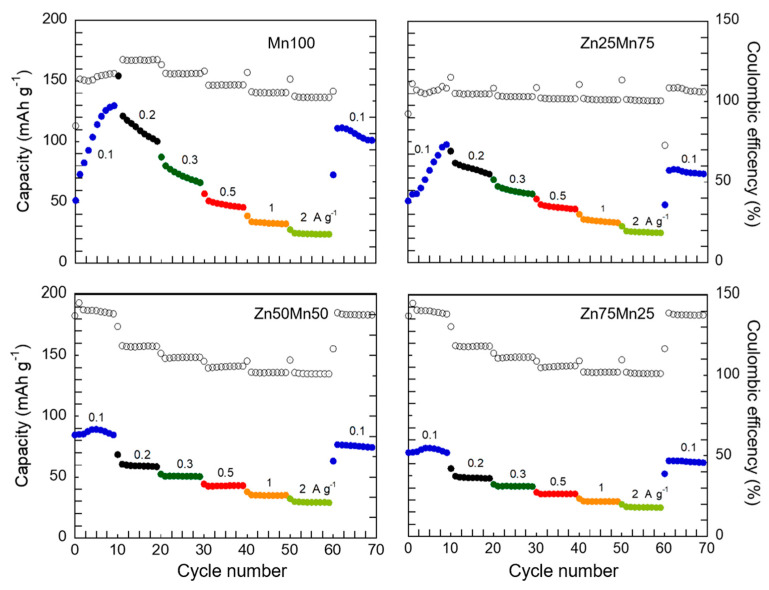
Rate performance and Coulombic efficiencies (open dots) of Mn_1−x_Zn_x_HCF (x = 0, 0.25, 0.5 and 0.75) samples.

Galvanostatic discharge and charge profiles at different current densities, i.e., 0.1, 0.2, 0.3, 0.5, 1.0, and 2.0 A g^−1^ are shown in Figure S5. As has been pointed out, at high current densities the capacity of the system decays, due to the limitations imposed in diffusion and charge transfer processes [63]. These limitations also cause the polarization to grow. This increase in polarization is more evident in samples with higher Mn content, which can be attributed to a higher difficulty of the cubic structure to facilitate the diffusion of Zn^2+^ ions. The comparison of the rate performance for the different materials analyzed in this work is shown in Figure 6. 

In terms of capacity, the values delivered by the Mn100 sample are higher than in the case of the Zn-doped phases, again, which is related to the higher amount of electrochemically active metals in Mn-rich phases. An increase in capacity is observed in the first cycles at 0.1 A g^−1^, being more critical for samples with higher Mn content. This effect in PBAs applied to AZiBs has already been reported in the literature [27,56] and is related to the structural readjustment of this type of compounds due to the need to deintercalate K^+^ cations. This process occurs in the first cycles, facilitating higher intercalation of Zn^2+^ ions as K^+^ leaves the structure. This structural conditioning process requires a series of cycles to occur, reaching maximum a capacity around the tenth cycle. Instead, in the case of Zn-enriched PBAs (Zn50Mn50 and Zn75Mn25 samples), the variation in the capacity values in the first cycles is less notable, due to the stability conferred on the structure by the presence of high amounts of zinc. The mechanism associated with this electrode activation process involves the release of K^+^ ions from the initial PBA that will be replaced by Zn^2+^ ions throughout the cycles. As has been demonstrated by theoretical calculations [64], in the initial phase due to de presence of K^+^, the MnN_6_ octahedra presents a moderate distortion of the structure due to the Jahn-Teller effect of trivalent manganese. The introduction of Zn^2+^ ions into the structure during the first cycles induces a much more intense Jahn–Teller effect that causes uncontrolled phase transitions in the material. This effect is more obvious the greater the amount of manganese present in the compound, so as the position of manganese is doped with zinc, the consequences of the structural distortion induced by the Jahn-Teller effect become less intense (since a smaller amount of Mn(III) exists in the compound) until it practically disappears for the Zn75Mn25 phase. Regarding the impact of the current intensity, the loss of capacity experienced by the samples when increasing the current is smaller when increasing the zinc content. In the case of Mn100 and Zn25Mn75, in the first cycles at 0.1 A g^−1^, the capacity increases considerably, reaching a maximum, that is comparable or even higher to other PBA families [23,25,64,65]. 

**Figure 7:**
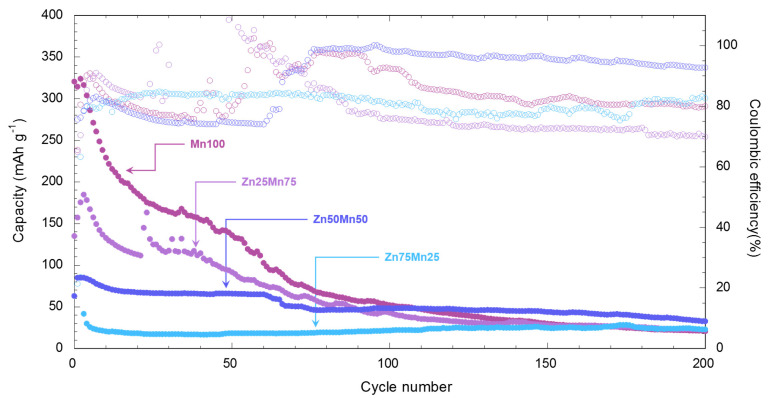
Long-term cycling of Mn_1−x_Zn_x_HCF (x = 0, 0.25, 0.5 and 0.75) samples at 50 mA g^-1^. The open dots refer to the Coulombic efficiency.

In order to corroborate the effect of zinc doping of the MnHCF phase on structural stability, cycling tests for 200 cycles have been carried out applying a current intensity of 50 mA g^−1^ (Figure 7). 

The low initial efficiencies are related to the irreversible insertion of Zn^2+^ ions, as has been reported by other authors [64]. The Mn100 sample presents the highest capacity but by cycle 20 it has already been reduced practically by half (184.7 mAh g^−1^) and continues to decay steadily until the end of the experiment (21 mAh g^−1^ in cycle 200, which represents only 6.5% of the initial capacity of 323 mAh g^−1^). As zinc replaces manganese in the MnHCF phase, a lower initial capacity is obtained related to a lower concentration of electrochemically active metals (Mn and Fe), as has been mentioned previously and described by other authors in similar studies [64,66]. In the case of zinc-rich phases, a lower effect of the electrode activation process and greater stability in terms of capacity can be seen. For the Zn50Mn50 sample, the capacity stabilizes at 70 mAh g^−1^ (82.6% retention of initial capacity of 84.7 mAh g^−1^) starting from cycle 10. This capacity value is maintained until cycles 60–65, where an abrupt drop occurs, up to 48 mAh g^−1^ which represents 56.7% of the initial capacity. The Zn75Mn25 sample suffers a drastic drop in capacity in the first 5 cycles, stabilizing around 23 mAh g^−1^ (55.3% initial capacity retention of 41.6 mAh g^−1^) throughout the entire cycling test. The manganese-rich samples, Mn100 and Zn25Mn75, exhibit a severe loss of capacity in the first 60–70 cycles, reaching capacities of 21 and 23 mAh g^−1^ in cycle 200, respectively (6.5% and 13.14% initial capacity retention). This significant loss of capacity is related to the dissolution of manganese in the aqueous medium of the electrolyte. Although the MnHCF phase presents great stability in an organic medium, it tends to undergo dissolution reactions of manganese ions in the electrolyte [24]. As a consequence of these processes, δ-MnO_2_ is formed, which is capable of reacting in the presence of protons, reducing to MnOOH, which in turn can be reduced in the aqueous medium to produce Mn^2+^ ions [13,67], through the following reactions:

MnO_2_ + H^+^ + e^−^ → MnOOH (2)

MnOOH + 3 H^+^ + e^−^ → Mn^2+^ + 2 H_2_O (3)

The loss of manganese ions in the PBA produces the formation of a zinc-rich phase that has a structure similar to that of the K_2_Zn_3_[Fe(CN)_6_]_2_ phase. In order to delve deeper into the stability of the compounds, a study using X-ray diffraction and scanning electron microscopy has been carried out on the electrodes once cycled. The SEM images and the diffractograms recorded after the long-term cycling experiment are shown in Figures S6 and S7. SEM analysis has been carried out to identify changes in morphology in the active material. In the images in Figure S6 it can be seen that the morphology of the materials is similar for all the electrodes, showing the presence of fibres that are remains of the glass fibre separator. Regarding the structural study, the manganese-rich samples (Mn100 and Zn25Mn75) undergo a clear structural change after cycling (Figure S7). The most characteristic peak of the cubic phase disappears and a peak at 24.33° (2θ) emerges with maximum intensity. The new diffraction profile fits with manganese oxohydroxide (MnOOH, JCPDS#41-1379) along with traces of the cubic phase (see Figure S8), which corroborates the dissolution process of manganese throughout the cycling. In this way, the degradation of performance is associated with the loss of active material. This dissolution reaction is less evident as zinc is introduced in the manganese position of the PBA, where the peaks corresponding to the initial compound are more visible. The post-mortem analysis corroborates that the introduction of zinc in the manganese sites of the MnHCF provides greater stability to the structure, preventing the insertion and extraction of Zn^2+^ ions in the battery cycle from inducing a phase transformation in the active material.

The analysis of the electrochemical response of the different materials presented in this work shows that zinc doping of manganese hexacyanoferrate provides greater stability to the material, causing, in turn, a loss in specific capacity. These results indicate that these compounds allow the reversible insertion/extraction of Zn^2+^ ions, without observing degradation upon cycling. Zinc doping is an easy strategy to implement to produce more stable cathode materials, although it is necessary to balance the amount of zinc to avoid a drastic loss of AZiB capacity.

## 4. Conclusions

In this study, the synthesis, physico-chemical characterisation and electrochemical study of the Mn_1−x_Zn_x_HCF (x = 0, 0.25, 0.5, 0.75 and 1) Prussian Blue analogues as cathodes for rechargeable aqueous Zn-ion batteries has been carried out. Substituting manganese with zinc induces a change in the structure, and a secondary rhombohedral phase appears accompanying the predominant cubic structure. This distortion in the phases has also been corroborated by infrared spectroscopy. The introduction of zinc into the MnHCF structure produces a change in morphology from a cubic to a cuboctahedral shape, which could translate into a larger specific area. This structural and morphological change has a direct impact on the electrochemical response of the compounds. Pristine MnHCF compound provides a high capacity of 130 mAh g^−1^ at 0.1 A g^−1^, but upon cycling this capacity is quickly lost due to the inherent weak structural stability of the compound. The instability of the Mn-rich phases has been corroborated through post-cycling studies, which demonstrate that manganese is susceptible to dissolving throughout the charge/discharge cycles, giving rise to the formation of the MnOOH compound. The impact of zinc substitution in manganese-based PBAs causes a decrease in the electrochemical activity, which is mainly due to the Mn^3+^/Mn^2+^ redox couple. Despite decreasing the specific capacity of the system, increasing the amount of zinc improves capacity retention and Coulombic efficiency. This way, it is proven that through a simple approach, by zinc doping, it is possible to obtain higher reversibility and stable performance for the MnHCF phase.

## Figures and Tables

**Table 1:** Syntheses that were carried out in the work.

Sample	ZnSO_4_ (mol)	Mn(NO_3_)_2_ (mol)	K_3_Fe(CN)_6_ (mol)	Theoretical Ratio (Zn/Mn)
Zn100	0.002	-	0.002	100:0
Zn75Mn25	0.0015	0.0005	0.002	75:25
Zn50Mn50	0.001	0.001	0.002	50:50
Zn25Mn75	0.0005	0.0015	0.002	25:75
Mn100	-	0.002	0.002	0:100

## Data Availability

The data presented in the study are available from the corresponding author.

## Supplementary Materials

The following supporting information can be downloaded at: https://www.mdpi.com/article/10.3390/nano14131092/s1, Figure S1: Schematic representation of the experimental setup for the synthesis of materials by the co-precipitation method; Figure S2: CV curves of the Zn100 sample at different scan rates: 1, 2, 5 and 10 mV s^−1^; Figure S3: Randles-Sevcik Plot for the Mn_1−x_Zn_x_HCF (x = 0, 0.25, 0.5, 0.75) samples; Figure S4: Discharge/charge profiles of the Mn_1−x_Zn_x_HCF (x = 0, 0.25, 0.5, 0.75) materials at a current density of 0.1 A g^−1^ in the 0.005–2.0 V vs. Zn^2+^/Zn range; Figure S5: Galvanostatic charge–discharge voltage profiles at different applied currents of the Mn_1−x_Zn_x_HCF (x = 0, 0.25, 0.5, 0.75) materials; Figure S6. SEM images of the Mn_1−x_Zn_x_HCF (x = 0, 0.25, 0.5, 0.75) electrodes after cyclability tests: (a,b) Zn75Mn25, (c,d) Zn50Mn50, (e,f) Zn25Mn75, and (g,h) Mn100; Figure S7. XRD patterns of the Mn_1−x_Zn_x_HCF (x = 0, 0.25, 0.5, 0.75) samples recorded after the synthesis (raw material), of the electrode before the electrochemical study (pristine) and of the post-mortem electrode after the cyclability study (post-cycling); Figure S8. Le Bail profile fitting of the powder X-ray diffraction pattern corresponding to the Zn25Mn75 electrode after cycling test.
