# Supplementary material for: Exploring Zinc-Doped Manganese Hexacyanoferrate as Cathode for Aqueous Zinc-Ion Batteries
Source: Nanomaterials (Basel). 2024 Jun 25;14(13):1092. doi: 10.3390/nano14131092 (PMC11243504; doi:10.3390/nano14131092)
Supplement: Supplementary file 1 [file nanomaterials-14-01092-s001.zip › nanomaterials-3051568-supplementary.pdf]

Exploring Zinc-Doped Manganese Hexacyanoferrate as Cathode for Aqueous Zinc-Ion Batteries

Julen Beitia [1], Isabel Ahedo [1], Juan Ignacio Paredes [2], Eider Goikolea [1] and Idoia Ruiz de Larramendi [1,*]

[1] Departamento de Química Orgánica e Inorgánica, Universidad del País Vasco (UPV/EHU), Barrio Sarriena s/n, 48940 Leioa, Spain; julen.beitia@ehu.eus (J.B.); isabel.ahedo@ehu.eus (I.A.); eider.goikolea@ehu.eus (E.G.)

[2] Instituto de Ciencia y Tecnología del Carbono, INCAR-CSIC, C/Francisco Pintado Fe 26, 33011 Oviedo, Spain; paredes@incar.csic.es

* Correspondence: idoia.ruizdelarramendi@ehu.eus

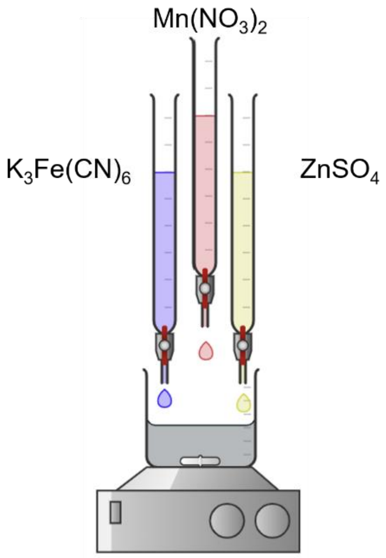

Figure S1. Schematic representation of the experimental setup for the synthesis of materials by the co-precipitation method.

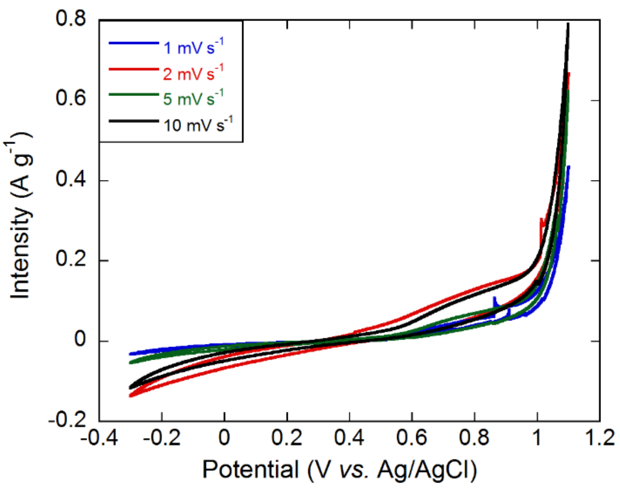

Figure S2. CV curves of the Zn100 sample at different scan rates: 1, 2, 5 and 10 mV s^{-1}.

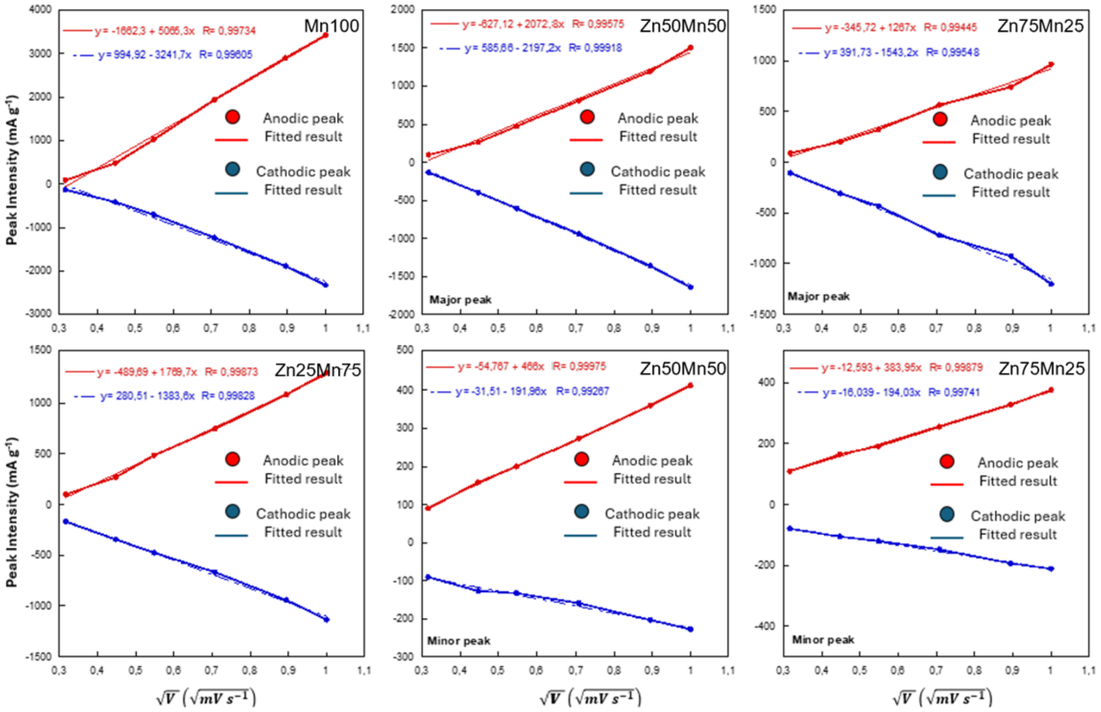

Figure S3. Randles-Sevcik plot for $Mn_{1-x}Zn_xHCF$ (x = 0, 0.25, 0.5, 0.75) samples.

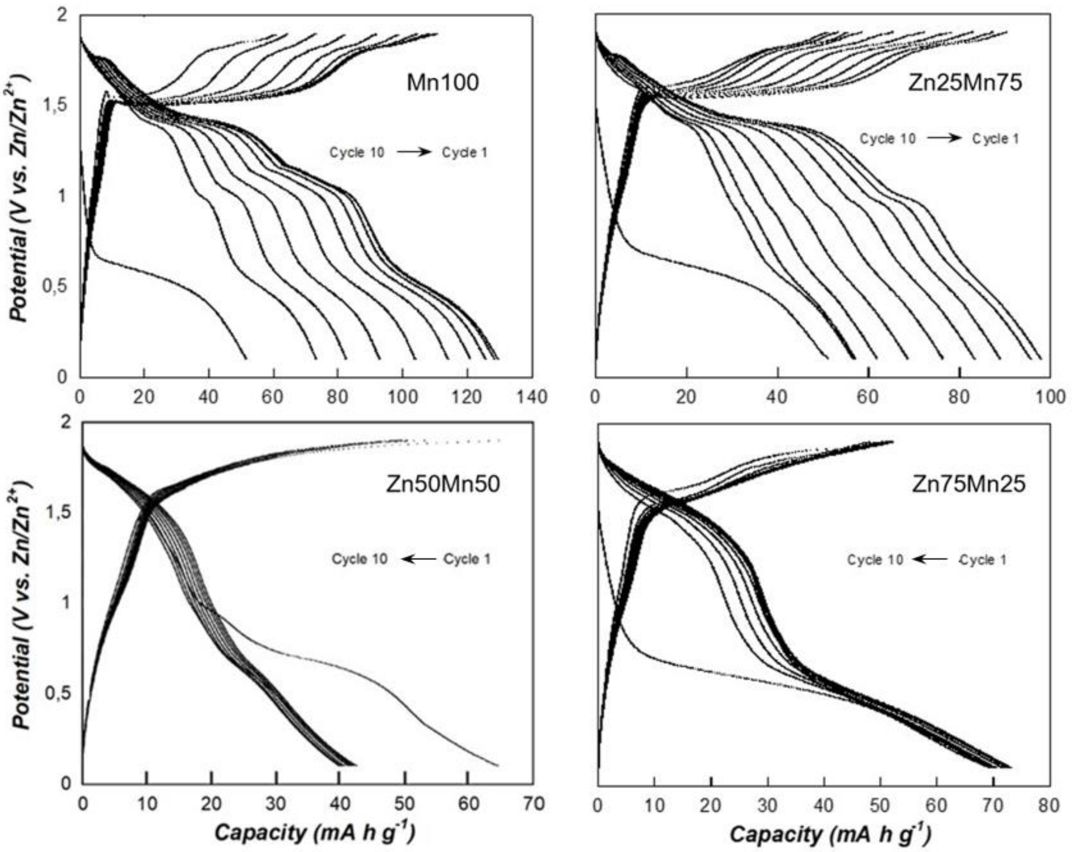

Figure S4. Discharge/charge profiles of the $Mn_{1-x}Zn_xHCF$ (x = 0, 0.25, 0.5, 0.75) materials at a current density of 0.1 A g^{-1} in the 0.005 – 2.0 V *vs.* Zn^{2+}/Zn range.

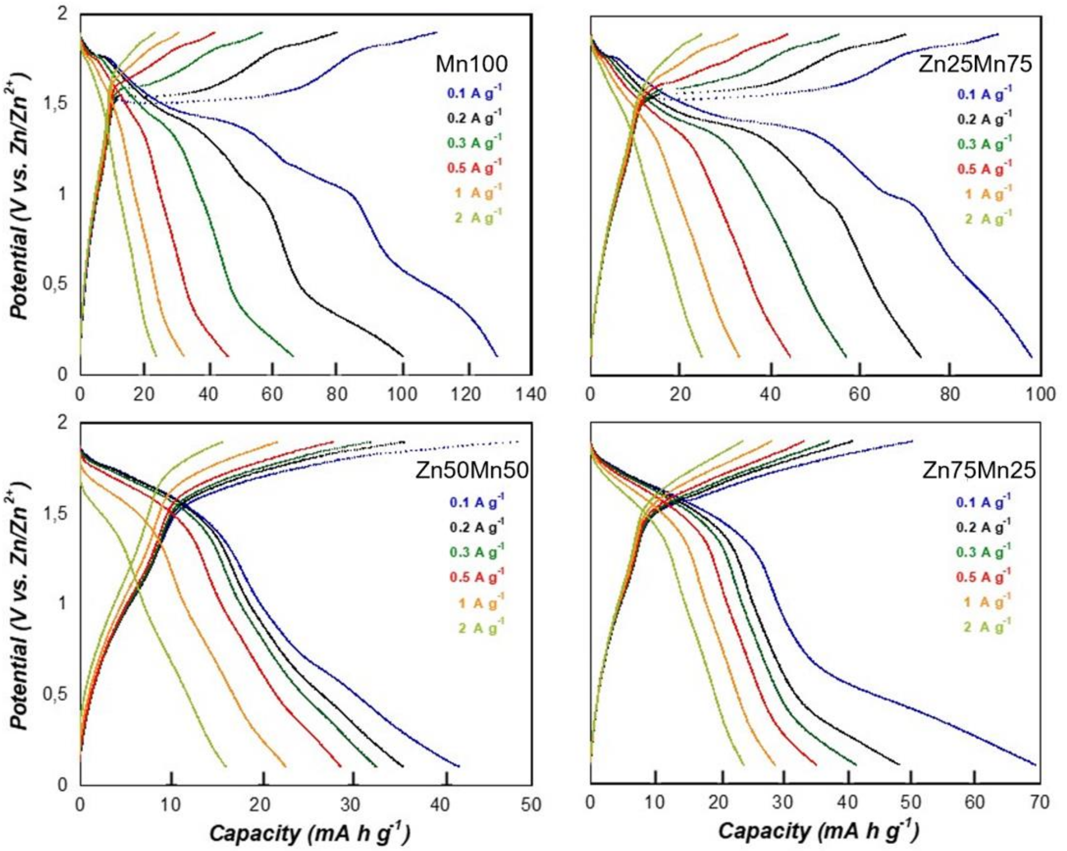

Figure S5. Galvanostatic charge–discharge voltage profiles at different applied currents of $Mn_{1-x}Zn_xHCF$ (x = 0, 0.25, 0.5, 0.75) materials.

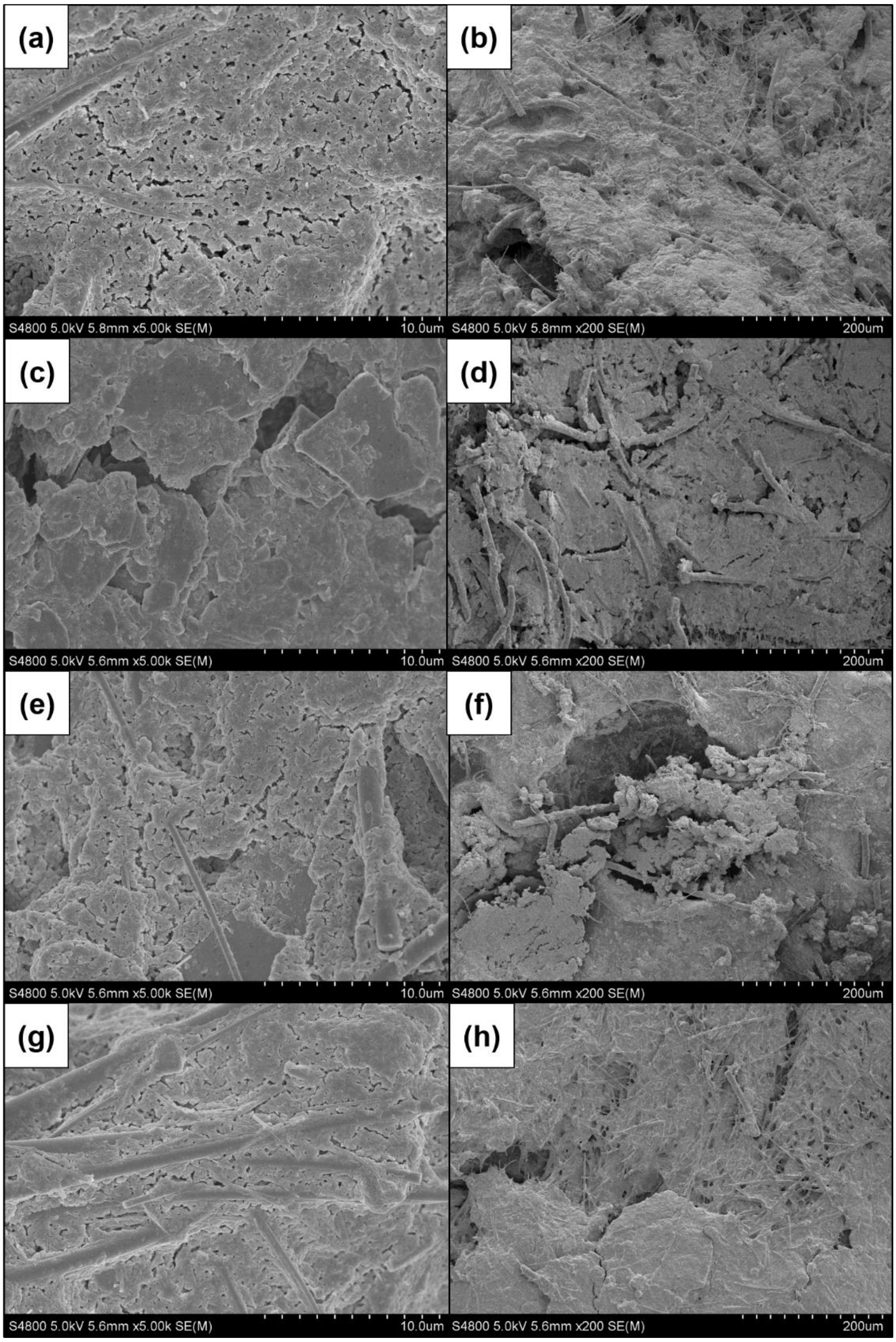

Figure S6. SEM images of the $Mn_{1-x}Zn_xHCF$ (x = 0, 0.25, 0.5, 0.75) electrodes after cyclability tests: (a,b) Zn75Mn25, (c,d) Zn50Mn50, (e,f) Zn25Mn75, and (g,h) Mn100.

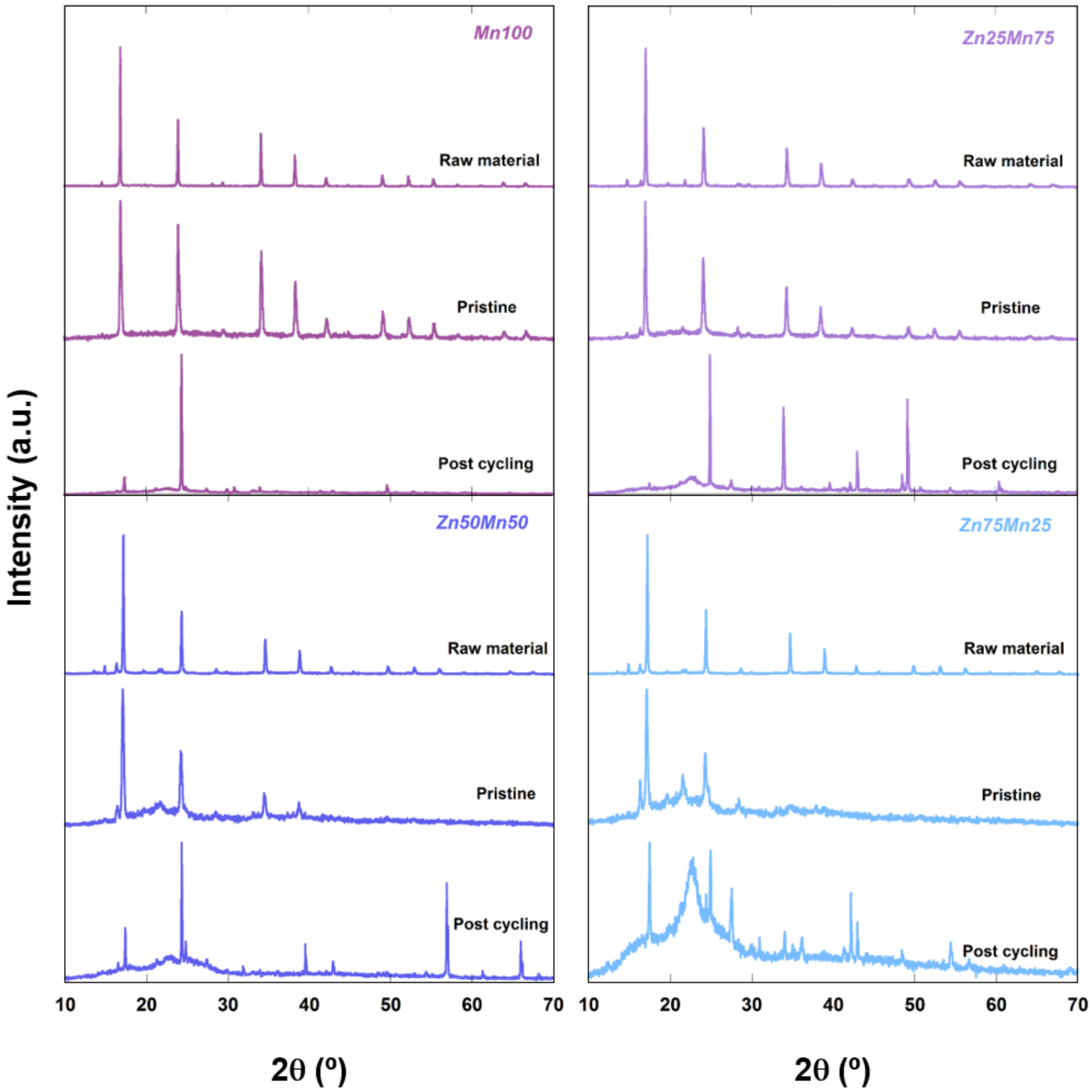

Figure S7. XRD patterns of the $Mn_{1-x}Zn_xHCF$ (x = 0, 0.25, 0.5, 0.75) samples recorded after the synthesis (raw material), of the electrode before the electrochemical study (pristine) and of the post mortem electrode after the cyclability study (post-cycling).

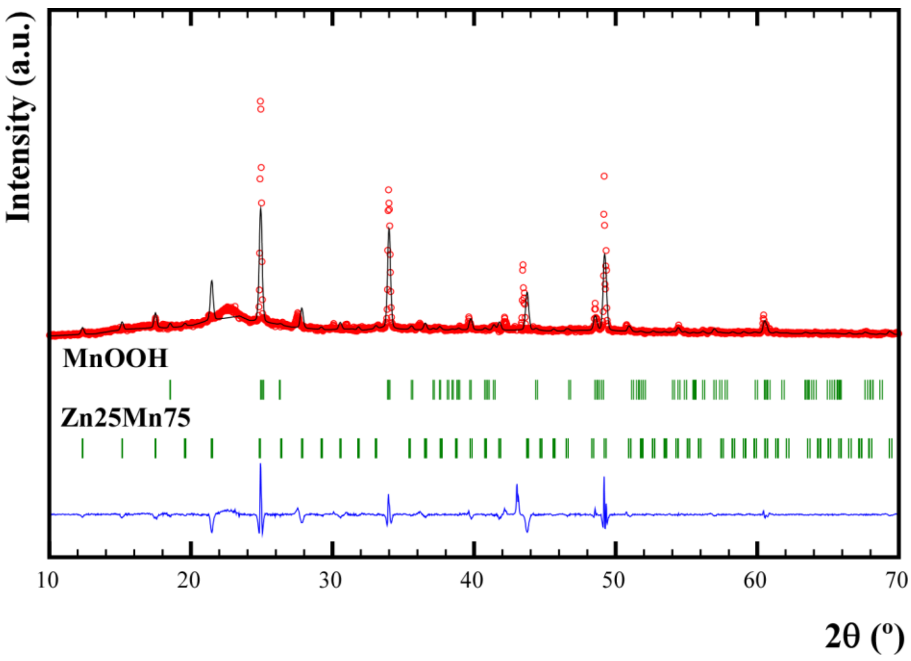

Figure S8. Le Bail profile fitting of the powder X-ray diffraction pattern corresponding to the Zn25Mn75 electrode after cycling test.
